# Enhancing Driver Monitoring Systems Based on Novel Multi-Task Fusion Algorithm

**DOI:** 10.3390/s25216799

**Published:** 2025-11-06

**Authors:** Romas Vijeikis, Ibidapo Dare Dada, Adebayo A. Abayomi-Alli, Vidas Raudonis

**Affiliations:** 1 Department of Automation, Faculty of Electrical and Electronic Engineering, Kaunas University of Technology, 51367 Kaunas, Lithuania; vidas.raudonis@ktu.lt; 2 Department of Computer and Information Science, Covenant University, Ota 112104, Nigeria; ibidapo.dada@covenantuniversity.edu.ng; 3 Department of Computer Science, Federal University of Agriculture, Abeokuta 110124, Nigeria; 4 Department of Software Engineering and Information Systems, Federal University of Agriculture, Abeokuta 110124, Nigeria; abayomiallia@funaab.edu.ng; 5 Institute for Systems and Computer Engineering, Technology and Science (INESC TEC), 4200-464 Porto, Portugal

**Keywords:** driver activity recognition, driver monitoring, driver attention analysis, multi-perspective learning, multi-task fusion, computer vision, deep learning, video classification, road safety

## Abstract

**Highlights:**

**What are the main findings?**

- A multi-perceptive and multi-task driver monitoring model is developed.
- A multi-task fusion algorithm for determining if the driver is attentive enough to drive safely is developed.

**What are the implications of the main findings?**

- Improved distraction detection accuracy: The multi-perspective monitoring approach outperforms traditional single-perspective systems, providing a more comprehensive and accurate assessment of driver tasks being performed and attention level.
- Real-time aggregation and decision-making: The multi-task fusion algorithm allows for stable and adaptive driver distraction classification, reducing false positives and improving response accuracy.

**Abstract:**

Distracted driving continues to be a major contributor to road accidents, highlighting the growing research interest in advanced driver monitoring systems for enhanced safety. This paper seeks to improve the overall performance and effectiveness of such systems by highlighting the importance of recognizing the driver’s activity. This paper introduces a novel methodology for assessing driver attention by using multi-perspective information using videos that capture the full driver body, hands, and face and focusing on three driver tasks: distracted actions, gaze direction, and hands-on-wheel monitoring. The experimental evaluation was conducted in two phases: first, assessing driver distracted activities, gaze direction, and hands-on-wheel using a CNN-based model and videos from three cameras that were placed inside the vehicle, and second, evaluating the multi-task fusion algorithm, considering the aggregated danger score, which was introduced in this paper, as a representation of the driver’s attentiveness based on the multi-task data fusion algorithm. The proposed methodology was built and evaluated using a DMD dataset; additionally, model robustness was tested on the AUC_V2 and SAMDD driver distraction datasets. The proposed algorithm effectively combines multi-task information from different perspectives and evaluates the attention level of the driver.

## 1. Introduction

The automotive industry is bigger than ever, counting 1.446 billion vehicles worldwide [1] and 252.6 million only in Europe [2]. Despite advances in vehicular systems, safety on the road remains a global issue. According to statistics, approximately 1.19 million people are killed in road traffic crashes each year (about 3200 people per day). Between 20 and 50 million people suffer non-fatal, yet very serious consequences from accidents [3,4]. The leading cause (~90%) of traffic accidents is human fault, and 68% of them are caused by drivers being distracted [5]. Distraction can be considered as any activity that shifts the driver’s focus from the road and the primary activity of driving. The most common examples of distractions are using a phone, eating or drinking, searching for something in the car, adjusting the radio, or GPS systems. Improvements in the automotive industry, development of semi-autonomous vehicles, and Advanced Driver-Assistance Systems (ADAS) further highlight the importance of in-cabin monitoring. The increasing prevalence of distraction-related accidents necessitates effective solutions [6]. Precise, adaptive monitoring is needed to assess occupant states and activities, and developing systems that ensure continuous driver behavior and attention analysis aid in detecting distractions and is essential for mitigating risks and enhancing road safety [7,8].

The increasing interest in artificial intelligence and its domains such as deep and machine learning has resulted in various improvements in driver monitoring systems (DMSs) [9]. Deep learning (DL)-based algorithms have proven to be an excellent techniques for DMSs that ensure high performance and robustness, even when operating on big and complex models, using large datasets [10]. Existing driver monitoring methods are promising; however, they face real-world limitations, such as unpredictable environmental conditions, focusing on singular or multiple tasks, such as combinations of distraction activities, emotion, or fatigue using only one input camera perspective. These partial views of drivers’ activities alone often lead to incomplete assessments of driver attention [11,12]. Although single-perspective driver monitoring remains the most popular approach, multi-perspective monitoring is superior because it reduces blind spots, enhances recognition accuracy by providing more information from multiple angles, and offers more complete view of the driver’s state and the vehicle’s surroundings [13]. Additionally, systems that utilize multi-perspective information can cross-reference decisions using multiple camera predictions, which can mitigate false alerts and lead to more accurate assessments of drivers’ activities [14]. A combination of multi-task recognition helps fully understand the driver’s state and intentions. For example, when the driver changes gear, he usually does not look at the road and one hand is off the wheel, and by recognizing multiple actions at the same time, we understand that the driver acts normally and no alert is needed, but if you monitor only gaze, this task could be considered as dangerous. Thus, combined monitoring allows for an accurate assessment and confirms the genuine attention of the driver [15]. Multi-task and multi-perspective monitoring provides a more complete picture of a driver’s true engagement and allows ADAS systems to act more precisely. While multi-modal or multi-task fusion improves general situation understanding, it introduces complexities related to feature representation, synchronization, and computational efficiency. Addressing these challenges necessitates innovative optimization strategies for scalability and real-time applicability [16,17].

To solve part of the mentioned limitations, this study proposes a model that focuses on simultaneous multi-task recognition: monitoring 10 of the most common driver activities, 4 hands-on-wheel states, and 2 gazes in the road directions. The research aimed to bridge existing gaps of “hidden” visual information by recognizing the above-mentioned tasks using a multi-perspective video input. The proposed CNN-based driver monitoring model is designed for accurate and efficient driver behavior analysis that could be adaptable for usage in real time. In addition to that, we present a way to determine the driver’s attentiveness to driving state by fusing weighted predictions from the proposed multi-task and multi-perspective model. The key contributions of this study include the following:

- An enhanced CNN-based model for simultaneous multi-task recognition of driver actions, gaze allocation, and hand–wheel interactions using multi-perspective video inputs, capturing the driver’s full body, hands, and face.
- Evaluation of multi-task information integration using feature fusion techniques to classify driver attention.
- Assessment of the proposed model by completing a test phase using unseen portions of information. We used 10% of the DMD dataset that was not seen in the training and validation phases and completely new datasets (AUC_V2 and SAMDD) for testing.

The remaining parts of this paper are structured as follows: Section 2 presents the literature review. Section 3 outlines the research methodology. Section 4 discusses the results obtained, followed by an in-depth discussion of findings, limitations, and future research directions. The study concludes in Section 6.

## 2. Literature Review

Driver attention monitoring systems are critical tools in modern vehicles, developed to improve driver safety while minimizing the likelihood of potential accidents because of driver distractions [18,19,20]. Advancements in the field include incorporating artificial intelligence (AI) strategies with sensors, cameras, and computer vision to monitor and examine driver activities [21]. This section provides an overview of the existing literature that analyzes the topic.

### 2.1. Related Work on Driver Monitoring Methods

Generally, driver distractions are categorized into three groups [22], namely: (1) visual, when the driver shifts eyes from the road; (2) manual, when the driver takes one or both hands off the wheel; and (3) cognitive, when the driver’s focus is on a task that is the primary driving activity or an activity related to driving. A lot of these actions can belong to one or all of the groups above. For instance, while texting; the driver is not looking at the road, has only one hand on the wheel (the other holds the phone), and is focused on something else other than driving (composing a text on the phone). Various sensors have been explored to monitor driver distraction, including cameras, microphones, contact sensors, inertial measurement units, and electroencephalography [12,23,24,25,26]. However, camera-based sensors are the most popular [27] due to the information they can carry and because cameras are not intrusive sensors, so they do not bother the drivers while driving. Research with camera data has proposed either image-based [17,28,29,30] or video-based [28,31,32,33,34] models. Image-based distraction classifications rely only on a single capture, which is the main limitation for such models. This has led to increasing research interests adapting 3D approaches for analyzing video data: holistically speaking, it is much easier to understand driver distraction following a dynamic sequence rather than from a single shot [14].

The existing literature that describes camera-based driver monitoring approaches offers different techniques, from reinforcement learning [35], to vision transformers [36], to federated learning [37], to various CNN-based [11] classifiers. In all these, deep learning is one of the most popular approaches for driver behavior recognition and accident prevention [6,38,39,40].

Ref. [7] proposed a DL-based occupant monitoring system to detect passengers and objects. Refs. [41,42] focus on predicted takeover time (TOT) in autonomous vehicles for safe control transitions, while [43] showed an improvement in TOT prediction accuracy across various secondary driver activities using data augmentation. Ref. [41] proved that the incorporation of DL algorithms in driver attention detection results in optimal precision and accuracy, usually through training and learning complex driver activities. In [43], a hybrid network approach for driver activity recognition was employed using hierarchical recurrent neural networks combined with ResNet and inception models. Refs. [44,45] also proposed hybrid model architectures using conventional convolutional block attention module layers and a VGG16 model and CNN combined with Bi-LSTMs, respectively. Ref. [46] used a generic CNN-based algorithm for driver distractions and incorporated face detection, hand localization, and skin segmentation for better results. Ref. [47] proposed an embedded DL approach for real-time driver distraction detection using SqueezeNet 1.1. Ref. [48] experimented with a combination of a CNN and attention-based capsule (CapsNet), which resulted in high model performance. Standard CNN architectures demand high computational power, making them difficult to implement in low-latency applications [7]. DL models also struggle with issues such as poor data annotation [49], generalization limitations [12], and overfitting [14]. Driver state misclassification remains a concern [6], particularly in occluded or complex driving scenarios. Many models also lack real-world validation and practical implementation details [19]. To address these limitations, lightweight CNN architectures optimized for edge devices have been explored, leveraging techniques such as parameter sharing and depth-wise separable convolutions [50]. However, achieving a balance between model efficiency and performance remains a challenge [37].

### 2.2. Related Work on Multi-Task Models for Driver Monitoring

Research has shown that fusing in-cabin driver data from multiple sources plays a big role in accuracy and reliability in driver monitoring. Fusing visual data (e.g., person, driving a car) from different viewing angles or visual data with sensor data (e.g., steering grip) enables a more comprehensive understanding of driver behavior. While this approach effectively captures inter-modal relationships, it often increases computational complexity [50]. There are several works that try to address issues of driver monitoring task, using multi-task (when several tasks are combined together, e.g., driver actions, fatigue, gaze on road, hands-on-wheel detection, etc.), multi-perspective (when several camera angles for the same view are used), and multi-modal (when information from different sensors are combined) approaches. Ref. [29] introduced MELD3, an approach that combines multi-task learning (MTL) and ensemble learning (EL) for driver distraction detection. MTL enables information transfer between similar tasks using different camera perspectives (body, face, and hand), which allows for a more generalized and robust model, while EL focuses on connecting the outputs from different actions using a soft-voting mechanism. After obtaining three input images, the model returns a prediction of one of ten possible driver actions. While testing results are promising, real-time adaptability is not addressed, and the model is interested only in one driver task, not the full awareness of the situation. Ref. [38] proposed an efficient multi-modal driver action recognition system based on dual-feature shift (DFS) that incorporates RGB, IR, and depth input sources for vehicle cabin monitoring. The proposed method integrates complementary features across modalities through the execution of modality features and interaction trying to recognize four driver actions: eating, drinking, and opening or closing a laptop. To understand regular patterns and increase model efficiency, DFS proposed sharing feature-extracting stages among multiple modalities. Even though the approach is promising, improvements in accuracy and inclusion of additional driver distraction actions are needed for real-time adaptability. For driver monitoring, ref. [31] proposed comprehensive in-cabin monitoring by employing two camera perspectives, capturing the driver’s body and face and focusing on four tasks: distraction action, gaze direction, hands-on-wheel, and fatigue detection. In addition, that work proposed fused driver attention scoring mechanisms. Different neural networks are used for each task: an Inflated 3D CNN (I3D) for distraction, SqueezeNet for gaze and fatigue, and a 3D CNN for hands-on-wheel detection. The system uses optical flow from body and facial landmarks from a face perspective to overcome single-perspective limitations. Experimental results showed that the multi-task, multi-perspective system can improve detection accuracy; however, a multi-network approach for each task really increases computational complexity and hardens real-time adaptability. Ref. [51] proposed a low-cost system to recognize driver actions, such as safe driving, distraction, drowsiness, and smartphone usage, using a lightweight YOLOv8n model with GhostNet modules running on a (Raspberry Pi Zero 2 W (Raspberry Pi, Cambridge, UK). The model integrates a GPU-accelerated head pose estimation algorithm via OpenCL 1.2. The model uses IR videos from two camera perspectives. The system ran at 10 FPS and reached acceptable results, but it was tested on a relatively small dataset, limiting the model’s generalization, and the frame rate might be insufficient for real-time adaptability.

Even though deep and machine learning models are the most popular for driver monitoring, vision transformer networks have been explored for driver monitoring tasks. Ref. [36] proposed a multi-task Vision Transformer for Driver Distraction Detection (ViT-DD) framework that focuses on driver actions and driver emotion recognition, using two camera (body and face) perspectives. The model uses a teacher–student approach, where a separate emotion recognition model provides pseudo-labels to support training on unlabeled information. While testing model, it was noticed that detection accuracy decreased for certain distraction categories (e.g., phone call left or reaching behind) due to misleading emotion correlations and class imbalance. Ref. [52] proposed a vision-transformer-based method that combines multi-task recognition of distracted activities and emotional state, also using a two-camera perspective. The authors utilized the ViT-DD model [36] and tried to improve it by utilizing a weighted loss function that emphasizes emotional detection accuracy, as the emotional state of the driver is very important for safe driving. The model uses RetinaFace for face detection and PAZ for emotion labeling, and it feeds body-perspective and face-perspective images into a ViT encoder with separate output heads. Tests on the dataset significantly improved emotion recognition when compared to the ViT-DD model; however, there are still challenges in order to combine these two tasks for real-world implementation.

Existing studies on multi-task and multi-perspective models are trying to solve limitations to single-approach models, and existing studies [29,31,51,52] prove that multi-task and multi-perspective models can improve driver monitoring; however, there are still some. Our research goal is similar to the research in [31], and while this work showed promising results, we will try to address some limitations we see in the mentioned work and use one model for all tasks instead of three separate ones, use a video input instead of images, and try to address the real-time adaptability of our model. In achieving all this, our goal is to increase the model’s performance metrics.

### 2.3. Datasets for Driver Monitoring

Various datasets have been proposed for evaluating driver distraction models; however, not all of them represent real-life scenarios. The publicly available datasets for driver distraction recognition include State Farm Distracted Driver Detection (SFDDD) [53], American University in Cairo Distracted Driver Dataset (AUC-DDD_V1) [54] and its expansion version, AUC-DDD_v2 [46], Drive&Act [26], Multi-Modal Driver Alertness Dataset (MDAD) [55], 3MDAD [56], driver monitoring dataset (DMD) [49,57], Synthetic Distracted Driving (SynDD1) [58], and Singapore AutoManNTU distracted driving (SAM-DD) [59]. Table 1 summarizes features of available datasets for driver monitoring tasks, where RGB stands for Red, Green, Blue and IR for Infrared color models.

Among all available datasets, DMD stands out because it is the most comprehensive dataset: it offers three camera perspectives, capturing the driver’s face, body, and hands simultaneously, capturing various driver activities, and mimicking real-life scenarios [29]. This combination makes DMD a perfect fit for our model, where we try to mitigate existing DL model issues. Refs. [40,60] pointed out that using a comprehensive dataset for training is potentially more important than the model itself.

## 3. Proposed Methodology

This study proposes a system that monitors a driver’s actions from in-cabin camera video data to decide if the driver’s attention state is safe or not for driving using common driver distraction activities, the driver’s hand-and-wheel interactions, and the driver’s gaze direction. When we are talking about distraction actions, we assume that each action is a separate event and does not happen simultaneously with another action, e.g., a distracting action can be texting, drinking, or adjusting the radio separately, not at the same time. However, driver gaze and hand–wheel interactions happen at the same time as a distracted action, e.g., when the driver is reaching for the backseat, he is not looking at the road and most likely has only one hand on the wheel.

**Figure 1:**
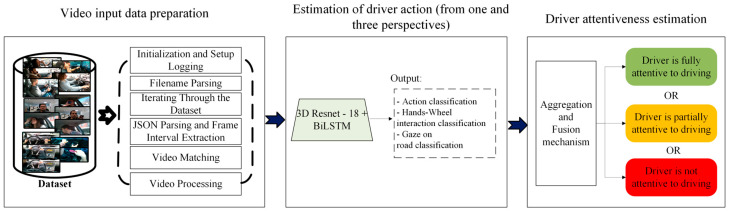
The flow diagram of the proposed approach.

Our proposed framework shown in Figure 1 consists of two parts: first, we propose a CNN-based model for individual task recognition using video input from three combined perspectives; the model gives outputs of the driver’s action, gaze directions, and hand–wheel interactions. The second phase is where the driver’s attention level to driving is determined using multi-task fusion. The proposed framework validation and testing experiments were carefully considered to make sure that the model is robust and reliable in real-world situations, where the model will face unseen situations, different people, etc. For this reason, we carried out the following:

- We trained and validated the model using 70% and 20% of the DMD dataset, respectively. The trained model was tested using a held-out corpus (10%) of the dataset. The drivers and data from this corpus were not used for model training or validation.
- In addition, cross-validation was performed with five folds.

These two steps give very small or zero overlap of the training and testing dataset splits and no perpetuation across the training, testing, and validating runs that were performed.

### 3.1. Data Preprocessing Pipeline

Video processing involves loading the video using video properties such as frame rate (fps), resolution, and total frame count. In addition, action interval processing is carried out for each action interval to check if the number of frames in the interval is less than 60; the interval is skipped or padded with the following frames based on the criteria. In addition, if there are remaining frames to/from a 60-frame-long clip, the video is padded with those following frames. If the clip is at the end of the video file with insufficient frames to be padded, it is skipped. The valid intervals are split into 60-frame segments, ensuring manageable clip sizes. Finally, video clip saving is performed for each 60-frame segment, saving as a new MP4 file in a subdirectory named after the action type (e.g., driver_actions/radio). Frames are resized to one-fourth of the original resolution before being written to reduce storage requirements while preserving expressiveness.

The outputs of the preprocessing include the processed clips that contain video clips of 60-frame segments for each action type, organized into folders by action class (e.g., /videos__driver_actions/radio), and the log file, which consists of a detailed log file documenting intervals skipped due to insufficient frames. This data processing pipeline effectively converts the annotated JSON data and video files into structured, action-specific training clips. The modular design and detailed logging make it a robust tool for preparing datasets for training machine learning models or conducting behavioral analysis.

### 3.2. Baseline Model and Its Evaluation

The system takes as the input videos categorized into three tasks, which include driver actions, consisting of videos capturing the physical actions performed by the driver, gaze on road, which consists of videos tracking whether the driver is looking at the road or not, and driver hand–wheel interactions, which monitor if the driver is holding the wheel. The feature extraction is a 3D Resnet-18 + BiLSTM-based model used for each task, as follows:

- Driver actions, where driver actions are classified into ten categories, such as safe driving, phone call left/right, reaching to the side, where a driver is looking to the side or trying to move his body towards the side while driving, reaching to the backseat, where a driver is checking his backseat while driving, texting left/right, attending to hair and makeup, using the radio, and drinking.
- Gaze detection, where gaze direction is classified into two categories: looking or not looking at the road.
- Hand–wheel interactions, where hand positions are classified into four categories: both, only left or right, or no hands on the wheel.

The proposed model was built and tested in three setups, which have slight differences between each other; however, the main architectures for task recognition are the same. The model combines a ResNet3D-18 backbone for spatio-temporal feature extraction with a ConvLSTM layer for temporal modeling, followed by a BiLSTM classifier head for final sequence classification. Different setups are explained below:

- Setup 1 produces seven models that were trained and validated on individual tasks from individual camera perspectives. For example, model 1 focuses on the body perspective and the driver’s action task, model 2 focuses on the body perspective and the hand–wheel interactions task, model 3 focuses on the body perspective and the driver’s gaze classification task, and so on. Each model of stage 1 focuses on a unique event from each camera perspective and gives one output at a time.
- In setup 2, three separate models were produced, using the same architecture as in setup 1, although instead of focusing on each task from one perspective, the model is trained to focus on individual tasks using a multi-perspective view. Each model of stage 2 focuses on an event from all camera perspectives, and similarly to stage 1, it predicts one output at a time.
- Setup 3, on the other hand, was trained on all camera perspectives and all tasks and has three outputs at a time, which consist of perspective (body, hands, or face camera), task (drive actions, hand–wheel interactions, gaze on road), and task category (safe driving, phone call left/right, reaching to the side/backseat, texting left/right, hair and makeup, using the radio, or drinking, and looking/not looking and one, both, or no hands on the wheel) for each task.

**Figure 2:**
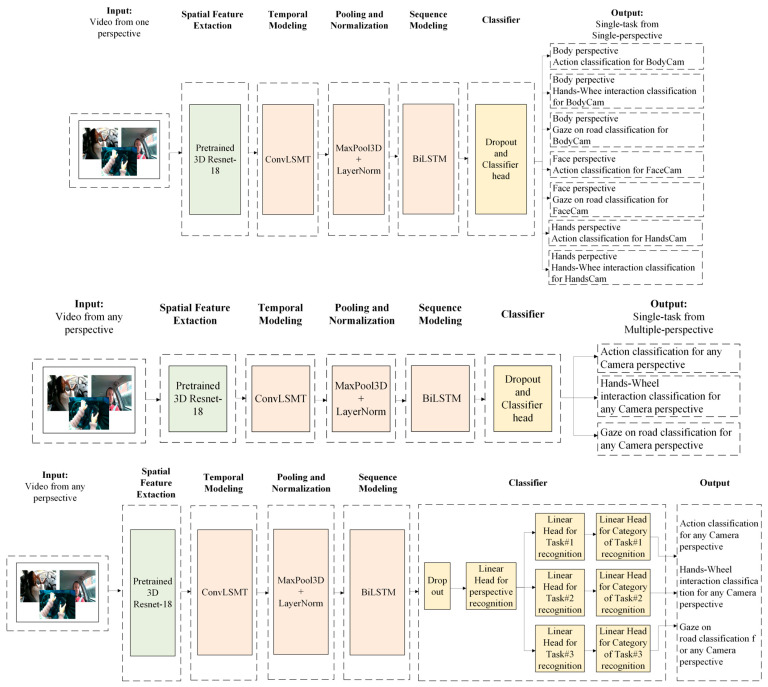
Proposed 3D Resnet-18 + BiLSTM model architecture for 3 setups: setup 1, on the top of the figure—separate perspectives and separate tasks; setup 2, in the middle of the figure—separate tasks but with combined perspectives; and Setup 3, at the bottom of the figure—one model for multi-task and multi-perspective recognition.

High-level model architectures for each setup are shown in Figure 2, and Table 2 provides a layer-by-layer summary of the proposed models.

The performance of the proposed models was evaluated based on the following standard metrics, which are accuracy, Equation (1), precision, Equation (2), recall, Equation (3), and F1-score, Equation (4).

Accuracy: The ratio of the correctly labeled samples to the whole set of samples.

(1) $$Accuracy=\frac{\left(TP+TN\right)}{\left(TP+TN+FP+FN\right)}$$

Precision: The ratio of the correctly labeled samples to the total number of correctly and incorrectly labeled videos.

(2) $$Precision=\frac{\left(TP\right)}{\left(TP+FP\right)}$$

Recall: The proportion of all actual positives that were classified correctly as positives, also known as the true positive rate.

(3) $$Recall=\frac{\left(TP\right)}{\left(TP+FN\right)}$$

F1-Score: The harmonic mean of precision and recall.

(4) $$F1\text{-}Score=\frac{2\times \;Precision\times Recall}{\left(Precision+Recall\right)}$$

ROC-AUC: The Area Under the Receiver Operating Characteristic (ROC) Curve measures the classifier’s performance and ability to distinguish between safe and unsafe states. A higher value indicates better performance.

### 3.3. Evaluation of Driver Attentiveness

Following the driver multi-task recognition evaluation in phase one and given its robustness and ability to generalize well across various driving conditions, the multi-perspective recognition model was integrated into the proposed multi-task fusion for driver attentiveness framework.

The proposed multi-task fusion framework fuses the driver’s actions, gaze, and hand position videos, integrates the outputs of the proposed model corresponding to each of the tasks, and prunes them into variant layers such as danger score computation, aggregation, and fusion, and finally, decision and alarm. This methodology is designed to ensure real-time reliable detection of distracted driving, providing an advanced safety mechanism for intelligent vehicle systems. The outputs of the multi-task multi-perspective ResNet-18 + BiLSTM model for each task are aggregated and fused to determine the driver’s overall safety state. Each classification output from the ResNet-18 + BiLSTM model is assigned a predefined danger score, as listed in Table 3, based on the risk level of the detected class.

The mathematical expression for computing the danger scores is calculated as shown in Equations (5)–(7).

(5) $$\mathrm{Driver}\;\mathrm{Actions}\;\mathrm{Danger}\;\mathrm{Scores}:\;S_{actions}=\sum_{i=1}^{n}P_{i}.D_{i}$$

(6) $$\mathrm{Gaze}\;\mathrm{Danger}\;\mathrm{Scores}:\;S_{gaze}=\sum_{i=1}^{n}P_{i}.D_{i}$$

(7) $$\mathrm{Hands}\text{-}\mathrm{on}\text{-}\mathrm{Wheel}\;\mathrm{Danger}\;\mathrm{Scores}:\;S_{hands}=\sum_{i=1}^{n}P_{i}.D_{i}$$

where $P_{i}$ is the probability of class, and $D_{i}$ is the predefined danger score for class  i. The aggregated danger score computation Equations (5)–(7) provide a structured method for assessing driver safety.

To set danger score values, research on the most dangerous activities while driving was conducted [31,61,62]. Ref. [61] states that even the smallest distraction can have critical consequences, where texting is the most alarming one, because while reading or writing a message, the driver is completely unaware as to what is happening on the road. The assignment of danger scores follows the ergonomic and human–machine interaction principles described in Standard ISO/TS 14198:2019 —Road Vehicles: Ergonomic aspects of the use of in-vehicle systems [62]. According to this standard, driver tasks must be judged based on their effect in terms of diverting visual, manual, or cognitive attention from the primary driving activity. Each activity class in Table 3 was mapped to a driver workload category, as specified in ISO/TS 14198, which quantifies secondary task intrusion.

Activities that require high visual and manual load simultaneously (e.g., texting or reaching for the backseat) are classified as Category 3 and assigned the maximum danger score (4–5). Tasks with moderate intrusion, such as drinking or reaching to the side, correspond to Category 2 and scores between 2–3. Finally, normal or low-interference behaviors (safe driving, both hands being on the wheel, looking at the road) correspond to Categories 0–1, with assigned danger scores of 0–1. This mapping provides an ergonomically justified and standardized explanation for the numerical danger weights, ensuring that the fusion algorithm’s risk assessment aligns with the established ISO-based evaluation of driver workload and attention demand.

The fusion mechanism is based on a danger score computation model, where each driver action, gaze state, and hand position is assigned a predefined risk level. The danger scores are aggregated using a weighted sum, Equation (8), where equal weight contributions are assigned by default to each task and perspective. The danger scores from the three tasks are fused to compute an overall risk assessment score, $S_{total}$, using the weighted sum approach.

(8) $$S_{total}=\frac{w_{1}.S_{actions}+w_{2}.S_{gaze}+w_{3}.S_{hands}}{w_{1}+w_{2}+w_{3}}$$

where $w_{1},{\;w}_{2},{\;w}_{3}$ are tasks weights (default: 1.0 for equal contribution). This fusion process enables a comprehensive evaluation of driver behavior by combining multiple cues, thereby reducing false positives and improving classification accuracy. This method ensures that all task categories and all perspectives contribute proportionally to the final driver safety state classification decision. To improve stability and reduce sudden fluctuations, a smoothing function, shown in Equation (9), applies a moving average window over the aggregated danger scores, enhancing robustness against transient distractions.

(9) $$S_{smooth}\left(t\right)=\frac{1}{k}\sum_{i=t-k+1}^{t}S_{total}\left(i\right)$$

If the smoothed danger score surpasses a predefined safety threshold $T_{safe}$, an alarm is triggered, Equation (10), alerting the system to a potential distraction or unsafe driving condition. This mechanism enables the real-time detection of unsafe driving behaviors, ensuring timely intervention to prevent potential accidents. The alarm system can be integrated into in-vehicle alert mechanisms, such as visual indicators, haptic feedback (steering wheel vibrations), or auditory warnings, providing an immediate response to detected distractions.

(10) $$A\left(t\right)=\{1,\;if\;S_{smooth}\left(t\right)>T_{safe}\;0,\;otherwise$$

**Figure 3:**
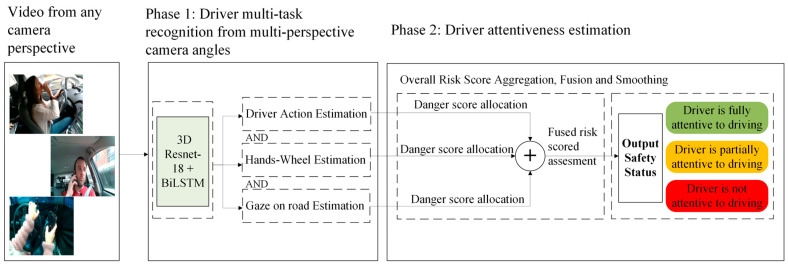
Multi-task fusion for driver attention and distraction using a multi-perspective driver task recognition framework.

The proposed model fusion and decision-making process enables real-time classification of driver states, making it a powerful tool for preventing distracted driving incidents. Figure 3 shows the proposed architecture for driver attentiveness level determination.

### 3.4. Technical Differentiation and Dynamic Feature Fusion Mechanism

Unlike traditional early- or late-fusion strategies, the proposed multi-task multi-perspective fusion framework incorporates feature representations dynamically at the decision level while maintaining cross-task contextual alignment. Approaches with early fusion usually use information of raw features before training, which can introduce redundancy and hinder task-specific learning [44]. Methods that use late fusion, on the other hand, concatenate outputs from independent tasks and use averaging or voting, which might lead to ignoring cross-task dependencies [38].

Our proposed framework introduces a dynamic feature selection mechanism, where the BiLSTM layer adaptively weights outputs from multiple perspectives (body, face, and hands) and multiple tasks (action, gaze, and hands-on-wheel). The weight coefficients are learned together through multi-task loss optimization, allowing the network to emphasize the most informative tasks under different conditions such as occlusion, pose change, or illumination differences. This structure allows for the adaptive contribution of each perspective to the final prediction, similarly to attention-based gating mechanisms, but this is optimized for multi-perspective driver monitoring, where information heterogeneity is high. The fusion of task features occurs in the latent space, Equation (8), before danger score computation, ensuring both multi-task synergy and multi-perspective consistency in the learned representation.

## 4. Experiments and Results

Following subsections describe the materials used for model creation and explains the results of the experiments.

### 4.1. Model Implementation and Experimental Setup

The model was built, trained, and tested using an NVIDIA A100 40 GB GPU with 83 GB of RAM (NVIDIA, Santa Clara, CA, USA). For model implementations, PyTorch 2.8.0 and CUDA 12.6 were used. Our model utilizes the cross-entropy loss (CELoss) function, Equation (11), which is commonly employed in classification tasks.

(11) $$Cross\text{-}Entropy=(1-\epsilon )ce(i)+\epsilon \;\sum_{i=1}^{N}\frac{ce(j)}{N}$$

where ce(i) is the cross-entropy loss for the true class; ϵ is the label smoothing factor, a parameter that controls the degree of smoothing; ce(j) are hyperparameters for the cross-entropy loss for each other class; and *N* is the total number of classes. Additional hyperparameters are listed in Table 4.

### 4.2. Dataset

For the proposed framework creation and evaluation, the driver monitoring dataset (DMD) was used [49,57]. The DMD dataset is one of the most comprehensive publicly available video datasets for driver monitoring. For DMD, video recordings were taken simultaneously from three different places inside of the vehicle, using body, face, and hand cameras and providing three different data formats: RGB, infrared, and depth. Videos were taken in two environments: in a real moving or stopped vehicle and in a simulator, along with different lighting conditions. In the data collection process, 30 drivers participated, and 10 of them wore glasses: 73% of them were men between 22 and 47 years old, and 27% were women between 21 and 38 years old. DMD provides distraction-, wheel-, gaze-, and fatigue-related activities. This data and the participant variety are very important for model robustness in real-world applications.

**Figure 4:**
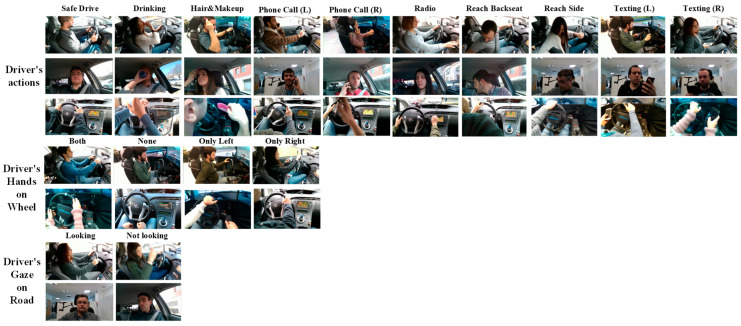
Sample of available snippets from the DMD dataset for each class.

For this paper, all available camera perspectives, from body, face, and hand cameras, were used, and they were categorized into three types: distraction-, hand–wheel-, and gaze-related. Figure 4 shows samples of available data from the DMD dataset from an RGB camera.

**Figure 5:**
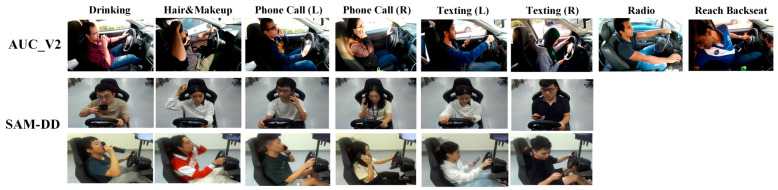
Sample of available snippets from the AUC_V2 and SAMDD datasets for the available classes.

Two publicly available datasets, AUC_V2 [46] and SAMDD [59], were used to test the model. The SAMDD dataset contains two perspectives, i.e., body and face cameras, while AUC_V2 consists of only the body camera perspective. Figure 5 shows samples from the AUC_V2 and SAMDD datasets. Table 5 summarizes the data samples used for model evaluation.

### 4.3. Results of Model Evaluation

In this study, training and validation were conducted using four different CNN architectures, i.e., 3D ResNet-50, 3D MobileNet_v2, 3D ResNet-18, and 3D ResNet-18 + BiLSTM, before deciding on the final backbone architecture. The models were trained to recognize three different tasks (gaze, hand–wheel interactions, and driver actions) from one perspective. Table 6 presents a summary of the training and validating results of each model, and Table 7 summarizes the validation-stage ROC AUC values per class for each of the three tasks.

From the results obtained, we concluded that even if all models showed good results, the 3D ResNet-18 + BiLSTM model performed the best in most of the cases. Due to this, it was decided to continue the model development and experiments with this architecture.

As the 3D Resnet-18 + BiLSTM model outperformed the other CNN models, all of the proposed models were tested in different setups. The initial experimental setup was carried out using the three different perspectives, resulting in the test results for the seven tasks, as depicted in Table 8. The second setup was based on combining different perspectives for each task. The overall results showed that the models could recognize the tasks using inputs from the multi-perspective cameras. Table 8 shows a summary of the separate task results obtained on testing setups 1 and 2.

Further validation on the trained DMD dataset was carried out by testing the model for its generalization capability on the test datasets (AUC_V2 and SAM-DD). Table 9 presents the summarized experimental results obtained by testing the proposed Resnet-18 + BiLSTM model on datasets other than the one it was trained on. The models reached accuracy values of 92.97%, 90.24%, and 89.19% on driver actions for DMD, AUC_V2, and SAM-DD, respectively. Accuracy values of 92.16% on gaze classification for DMD, 91.05% for AUC_V2, and 90.66% for SAM-DD were obtained. Accuracy values of 92.37%, 90.06%, and 89.77% were obtained for hands-on-wheel detection for DMD, AUC_V2, and SAM-DD, respectively.

**Figure 6:**
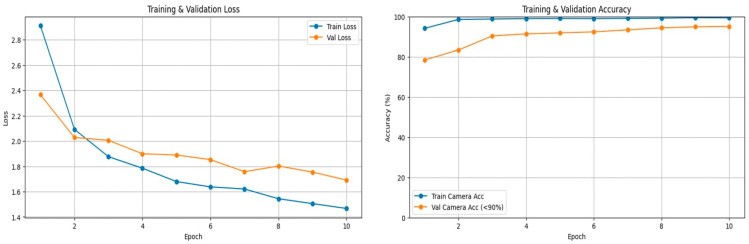
Training and validation accuracy and loss curves for the proposed multi-action and multi-perspective model.

**Figure 7:**
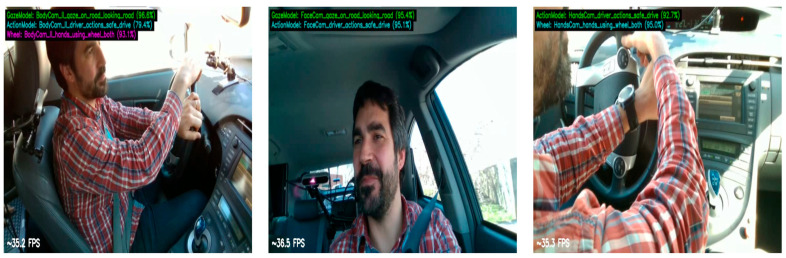
Example of testing outputs for multi-perspective, multi-task model at the same time point from testing videos from different perspectives.

After confirming that the multi-perspective models are robust in terms of recognizing separate tasks, the final multi-task multi-perspective model was build. The model reached an accuracy average of 93.74% on the testing dataset. Table 10 summarizes the multi-task multi-perspective model testing results. Figure 6 shows the training and validation loss and accuracy graphs. Figure 7 shows an example of the testing output of each camera perspective at the same given moment in time. The model recognizes which perspective the video input is from; the body camera perspective is on the left, the face camera perspective is in the middle, and hand camera perspective is on the right side of Figure 7. The model recognized three tasks at the same time for the body camera perspective (left) and predicted that the driver was 96.9% looking at the road, 93.1% having both hands on the wheel, and 79.4% that this was a safe driving action. For the face camera perspective, the model recognized two tasks at the same time, predicting that it was 95.4% looking at the road and 95.1% that the action was safe driving. For the hand camera perspective, the model predicted that 92.7% was safe driving and 95.0% was both hands using the wheel.

To understand how the final proposed model would perform on embedded or edge devices, market research was conducted, where it was found that, currently, the NVIDIA Jetson AGX Orin (NVIDIA, Santa Clara, CA, USA), is one of the best and had the most potential to be used in tasks such as driver monitoring [63,64]. The NVIDIA Jetson AGX Orin device has an 8 or 16 GB GPU with 32 GB or 64 GB of RAM, based on modification. We ran our model on an NVIDIA T4 16 GB GPU (NVIDIA, Santa Clara, CA, USA), that had 16 GB RAM. The average model inference was 20.48 ms per video input. The received results on the setup, similar to an edge device, were approximately 2 times slower, which is an acceptable result considering that this environment is much less powerful than a desktop-based setup.

The proposed model achieved results that are strong and can compete with existing state-of-the-art methods and in some ways to outperform them due to its multi-information usage. Section 4.4 compares proposed model with similar state-of-the-art research works.

### 4.4. Benchmarking Results Obtained Against Existing Studies on Driver Monitoring

Table 11 presents a comparison of the proposed method with existing state-of-the-art methods on the DMD dataset.

It is important to point out that it is not completely fair to compare all the methods with each other due to the different focus points of the studies and the different ways of using the same dataset. For example, the first difference is that the DMD dataset is originally a video dataset, but some studies [29,30,31,52] have converted it into images and focused on distraction recognition from one frame. Secondly, the DMD dataset is composed of videos from three different perspectives, i.e., body, face, and hands, but two of the mentioned studies, ours and [29], used all three available perspectives, refs. [31,52] used two available perspectives, and remaining works [30,32,33,34] used only one perspective. Moreover, the DMD dataset offers a variety of tasks for driver monitoring, such as action recognition, gaze and hand monitoring, or fatigue monitoring, but studies, including ours, did not utilize all tasks, and furthermore, each uses a very different number of task categories in their studies. The last thing that is important to mention is that studies used different methods for driver monitoring: some utilize transformers [32,34,52], some use ensemble learning [29], and some use DL methods [30,31,33], as well as the methods used in this study.

So, even though all studies have core differences, we see that our proposed method can compete with the methods in existing studies based on a more comprehensive and accurate assessment of driver attention monitoring.

To emphasize the benefits of the proposed dynamic multi-task, multi-perspective fusion, we conceptually compared it with traditional early- and late-fusion schemes. Standard early fusion joins raw features at the input stage, often leading to redundancy and noise, while late fusion incorporates final predictions, losing intermediate cross-task dependencies. In distinction, our dynamic mechanism performs mid-level feature weighting that adjusts to context and task relevance, allowing for improved discriminatory capacity across different driver states. Future work will extend this evaluation through controlled benchmarking against explicit early-/late-fusion baselines.

### 4.5. Results Obtained from the Driver Attentiveness Predicting Framework

**Figure 8:**
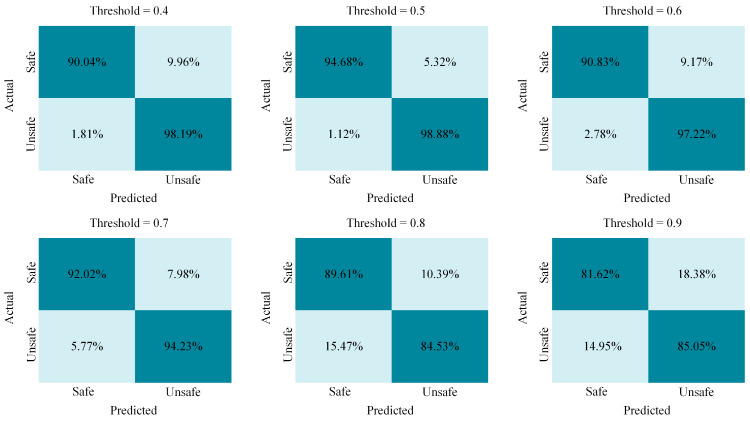
Summary of fused model confusion matrix for predicting driver attentiveness state on the DMD dataset across varying thresholds.

The danger score fusion mechanism combines the three tasks’ individual risk scores to determine the overall driver safety state. The weighted sum function in Equation (8) ensures that each task category contributes proportionally to the final safety score. A moving average function in Equation (9) smooths out fluctuations, making the proposed model less sensitive to momentary distractions, while Equation (10) triggers an alarm if the aggregated danger score exceeds a predefined threshold, ensuring immediate intervention in the case of unsafe behavior. Table 12 presents the performance of the fused model predicting the driver’s attentiveness state using different threshold values. Figure 8 shows confusion matrixes for each threshold.

The proposed driver attentiveness prediction model aims to successfully address some of the limitations in existing DMS by integrating multi-perspective and multi-task data, leveraging 3D ResNet-18 + BiLSTM’s performance and utilizing an optimized danger-score-based classification framework. From the reported results, it is possible to see that with a threshold of 0.5, the proposed multi-task fusion mechanism reached higher results in terms of recall and F1-score. From the confusion matrix in Figure 8, it can be deduced that with this threshold, the least false negative and false positive results are reported. A value of 0.5 is the optimal threshold for this experimental setup.

## 5. Discussion and Future Work

The proposed framework for driver task recognition and attentiveness to driving monitoring performs quite accurately within its configuration; however, there are places where it could be improved for better results and more precise estimation and adaptability for real-world usage under conditions that have different distraction categories. During our work, we focused on the main distracted driver actions, such as texting, making a call, drinking, adjusting the radio, attending to hair or makeup, reaching to the side or the backseat, the driver looking or not looking at the road, and hand–wheel interactions, such as the driver holding the wheel with both, one, or no hands. However, the variety of actions and distractions that a driver might experience in real life is huge and usually unforeseeable. For that reason, DMS need to be really sophisticated and recognize naturalistic safe and unsafe driver–vehicle interactions. Further potential improvements and tasks for future work are listed below.

**Expanding the list of driver tasks of interest**. The proposed model is capable of recognizing three tasks that happen simultaneously; however, categories of each task might be expanded in future work; for example, when our model categorizes a driver’s gaze on the road, it has only two categories: looking or not looking at the road, and in real-life scenarios, there are more gaze directions that are not considered dangerous, such as the driver looking to the rear or side mirrors before performing some maneuver. The same is true with a combination of driver actions and hand–wheel interactions; sometimes, one hand being on the wheel is not dangerous at all, like when the driver is changing gear. Thus, expanding the task categories would be beneficial for the model and its effectiveness across a wider number of scenarios. In addition, the accuracy might increase if the list of driver distraction actions were to be reviewed on each particular perspective; for example, it is very hard to determine if a driver is adjusting the radio or texting from the face camera, and so on.

**Testing the model in real-world applications**. The proposed model was built and evaluated using one of the most comprehensive publicly available driver monitoring datasets—DMD—and tested on other distracted driver datasets—AUC_V2 and SAMDD—which are also publicly available. Even though the testing results showed that the proposed model can recognize driver distractions quite well on unseen information, the performance of the model was not tested in real-world applications. So, testing the model in real-life environments using edge devices would be another future task to complete.

**Improving the model’s accuracy and adaptability to unseen scenarios.** While testing the proposed model on the AUC_V2 and SAMDD datasets, the accuracy dropped by about 2%. Future work could investigate this and train the model with more diverse datasets or adapt augmentation techniques to make the model more robust to unseen driver and environmental uncertainties, such as illumination variations and occlusions.

**Model optimization** could be another place for improvement. Making the model smaller but maintaining the same or obtaining even higher accuracy would be beneficial for model usage in devices with low power and limited computational resources. This would make our model more compatible with ADAS systems. Our model was tested on a “close to edge device specification” setup and reached an inference time of 20.48 ms for one input, and it outperformed the processing time of another work [36] by 7.52 ms. However, the decision-making time still can be improved in the future through model optimization.

**Network selection.** For the proposed model, we used the 3D-ResNet-18 + BiLSTM network. DL architectures are a very common selection for DMS due to their ability to recognize complex patterns in the provided data and due to them being highly effective for tasks like recognizing driver distractions, emotions, or drowsiness in real-time. However, the same idea of multi-task recognition using multi-perspective data sources can be adapted with other techniques. Ref. [65] provides a review of novel 3D object recognition methods in the perception frameworks of autonomous systems. With an emphasis on LiDAR–camera integration, ref. [66] introduces the Diffusion Model-Optimized Neural Radiance Field (DT-NeRF) method, which has the purpose of enhancing detail recovery and multi-view consistency in 3D scene reconstruction. The spatio-temporal feature soft correlation concatenation aggregation structure proposed in [67] might solve the issue that general models face while trying to recognize actions that might look similar, for example, adjusting the radio and setting the GPS system. The method proposed in [68] can be adapted while trying to solve low-lighting conditions and improve model lightness. The mentioned models were not adapted to automotive applications, but they have theoretical potential to improve ADAS systems. Future work could adapt one or all of the methods that are not so common in automotive applications.

In conclusion, even though there are places where our proposed framework can be improved, we do believe that in the current setup, our model could already be great addition to DMS, as it recognizes three driver tasks simultaneously from three different perspectives, having, in total, sixteen categories: ten categories for driver actions, such as safe driving, phone call left/right, reaching to the side or backseat, texting left/right, attending to hair and makeup, using the radio, and drinking; two for gaze detection, such as looking or not looking at the road; and four for hand–wheel interactions, such as both hands, no hands, or only the right or left hand on the wheel. In addition, we proposed a multi-task fusion algorithm to determine the state of the driver and alert if the driver’s state is not attentive enough to ensure safe driving.

## 6. Conclusions

This study proposed a multi-task fusion method for driver attentiveness and distraction analysis using multi-perspective data received from cameras placed at different places in the vehicle. The model leverages the architecture of 3D ResNet-18 combined with BiLSTM for driver behavior analysis. The proposed framework was evaluated across three key tasks: driver actions (with 10 actions), gaze detection (2 directions), and hand–wheel interactions (4 possible categories of hands on the wheel), using different setups for the integration of multi-perspective video data. The model was built and initially evaluated on the DMD dataset; in addition, the model was tested on another two datasets, AUC_V2 and SAMDD, to check the model’s performance on data that was not seen at any stages of model development. The proposed data fusion algorithm effectively combines different data sources and evaluates the danger level by a comprehensive aggregated danger score, and it is designed to enhance real-time driver monitoring and distraction detection.

The experimental results demonstrated that the proposed multi-task and multi-perspective model achieved 93.74% accuracy in multiple driver task recognition tasks at once and 96.78% accuracy in determining the driver’s attentive state based on three recognized driver tasks. These results validated that the proposed model has an optimal architecture for driver attention monitoring, providing a more accurate, scalable, and efficient solution for real-time driver safety assessment. A key contribution of this study is that the model that can handle information from different perspectives and make predictions on multiple tasks at the same time. A framework that aggregates different tasks, makes decisions, and incorporates an alarm trigger mechanism within the real-time environment is another key contribution of the proposed framework. This fusion-based decision-making process significantly enhances the reliability of driver safety predictions, making it suitable for real-time deployment in driver assistance systems.

The key findings and potential real-world implications of the study are as follows:

**Improved distraction detection accuracy**: The proposed model outperforms traditional single-perspective systems, providing a more comprehensive and accurate assessment of driver attention.

**Real-time aggregation and decision-making**: The danger score fusion and alarm trigger mechanism allow for stable and adaptive driver distraction classification, reducing false positives and improving response accuracy.

**Accident risk reduction**: By accurately identifying distractions in real time, this system can be integrated into vehicles to enhance safety and compliance with road regulations.

These findings contribute to advancements in AI-driven driver assistance technologies, paving the way for more intelligent, adaptive, and safer road environments.

## Figures and Tables

**Table 1:** Summary of available datasets for driver monitoring.

Dataset	Input Type		Video Stream				Perspectives			
	Image	Video	RGB	Grayscale	RGB and Depth	RGB and IR and Depth	1	2	3	6
SFDDD	x		x				x			
3MDAD	x					x		x		
MDAD		x				x		x		
AUC-DDD_V1		x	x				x			
AUC-DDD_V2		x	x				x			
SAM-DD		x			x			x		
DMD		x				x			x	
Drive&Act		x				x				x
SynDD1		x		x					x	

**Table 2:** Layer summary of proposed models.

**Setups 1 and 2**		
**Layer/Block**	**Input Shape**	**Output Shape**
3D-Resnet-18 Backbone	[1, 3, 16, 112, 112]	[1, 512, 1, 1, 1]
BasicStem (Conv + BN + ReLU)	[1, 3, 16, 112, 112]	[1, 64, 16, 56, 56]
Residual Blocks (x4)	[1, 64 → 512]	[1, 512, 2, 7, 7]
ConvLSTM	[1, 2, 512, 7, 7]	[1, 2, 128, 7, 7]
MaxPool3D	[1, 2, 128, 7, 7]	[1, 2, 128, 3, 3]
LayerNorm	[1, 2, 128]	[1, 2, 128]
Bi-LSTM (Hidden = 512)	[1, 2, 128]	[1, 2, 512]
Dropout	[1, 512]	[1, 512]
Classifier Head	[1, 512]	[1, 3]
**Setup 3**		
**Layer/Block**	**Input Shape**	**Output Shape**
3D-Resnet-18 Backbone	[1, 3, 16, 112, 112]	[1, 512, 1, 1, 1]
BasicStem (Conv + BN + ReLU)	[1, 3, 16, 112, 112]	[1, 64, 16, 56, 56]
Residual Blocks (x4)	[1, 64 → 512]	[1, 512, 2, 7, 7]
ConvLSTM	[1, 2, 512, 7, 7]	[1, 2, 128, 7, 7]
MaxPool3D	[1, 2, 128, 7, 7]	[1, 2, 128, 3, 3]
LayerNorm	[1, 2, 128]	[1, 2, 128]
Bi-LSTM (Hidden = 512)	[1, 2, 128]	[1, 2, 512]
Dropout	[1, 1024]	[1, 1024]
Linear Head (1–4)	[1, 1024]	[1, 3]
Linear Head (1–5)	[1, 1024]	[1, 3]
Linear Head (1–6)	[1, 1024]	[1, 16]

**Table 3:** Danger scores for each activity class.

Driver’s Action		Driver’s Gaze on the Road		Driver’s Hands on the Wheel	
Action	Danger Score	Gaze Direction	Danger Score	Hands on the Wheel	Danger Score
Texting (Left/Right)	5	Not Looking	3	None	5
Phone Call (Left/Right)	4				
Reaching to the Backseat	4				
Drinking	3			Only Left/Right	2
Reaching to the Side	2	Looking	0		
Hair and Makeup	1				
Radio	1			Both	0
Safe Driving	0				

**Table 4:** Hyperparameter values used for the development of the proposed model.

Hyper-Parameter	Value
Batch Size	32
Learning Rate	Starts at 0.0001 but is updated by the optimizer
Dropout Rate	0.3
Number of Epochs	10
Optimizer	Adam
Scheduler	lr_scheduler.cosineAnealing

**Table 5:** Summary of used datasets.

Dataset	Number of Videos	Purpose
DMD	7961	Training, validating, and testing
AUC_V2	100	Testing
SAM-DD	100	Testing

**Table 6:** Summary of CNN models’ performance for each task using one perspective view using the DMD dataset.

CNN Architecture	Accuracy, %		Precision, %		Recall, %		F1-Score, %	
	Training	Validation	Training	Validation	Training	Validation	Training	Validation
Gaze Classification								
3D ResNet-50	98.98	**97.63**	96.35	89.35	92.95	85.24	94.58	**97.17**
3D MobileNet_v2	96.80	96.28	87.06	81.76	76.55	77.77	80.83	77.77
3D ResNet-18	98.61	94.05	94.35	90.57	94.06	90.38	94.20	90.47
3D ResNet-18 + BiLSTM	**99.79**	97.04	**97.47**	**94.83**	**96.68**	**96.15**	**97.07**	95.49
Hand–Wheel Interactions								
3D ResNet-50	99.55	97.61	98.68	93.32	98.04	92.78	98.35	93.04
3D MobileNet_v2	98.20	97.02	96.07	93.26	92.71	90.48	94.20	91.71
3D ResNet-18	98.58	96.36	98.04	96.14	97.98	96.02	98.01	96.08
3D ResNet-18 + BiLSTM	**99.86**	**99.15**	**99.17**	**99.10**	**99.15**	**99.24**	**99.16**	**99.15**
Driver Actions								
3D ResNet-50	99.94	**98.85**	**99.93**	98.04	**99.93**	**97.77**	**99.93**	97.90
3D MobileNet_v2	98.47	97.59	98.07	96.28	96.69	94.42	97.33	95.18
3D ResNet-18	98.87	97.66	97.30	96.74	96.89	95.43	97.09	96.08
3D ResNet-18 + BiLSTM	**99.96**	98.44	98.61	**98.47**	98.44	97.21	97.87	**98.44**

**Table 7:** Summary of CNN models’ validation-stage ROC AUC values per class for each of the three tasks.

Task	Class	3D ResNet-50	3D MobileNet_v2	3D ResNet-18	3D ResNet-18 + BiLSTM
	Safe Driving	0.9997	0.9991	**1**	**1**
	Phone Call (Left)	**1**	0.9998	**1**	**1**
	Phone Call (Right)	**1**	0.9999	**1**	**1**
**Drivers**	Reaching for the Side	**0.9999**	0.9997	0.9998	**0.9999**
**Actions**	Texting (Left)	**1**	0.9999	0.9997	0.9998
	Texting (Right)	**0.9999**	0.9997	0.9998	**0.9999**
	Hair and Makeup	0.9999	0.9993	**1**	**1**
	Radio	0.9999	0.9999	**1**	**1**
	Drinking	0.9999	0.9979	0.9999	**1**
	Reaching for the Backseat	**0.9999**	0.9959	0.9964	**0.9999**
**Driver’s Gaze on the Road**	Not Looking	0.9596	0.9371	0.9441	**0.9693**
	Looking	0.9596	0.9371	0.9441	**0.9693**
**Driver’s Hands on the Wheel**	Both	0.9983	0.9985	**0.9999**	0.9993
	Ony Left	0.9983	0.9987	**1**	**1**
	Only Right	0.9993	0.9986	**1**	**1**
	None	0.9742	0.9670	0.9999	**1**

**Table 8:** Summary of proposed model performance in testing stage for each setup.

	Task	Accuracy, %	Precision, %	Recall, %	F1-Score, %	Inference Time, ms
**Setup 1**						
Body Camera Perspective	**Gaze Classification**	93.33	93.47	93.33	93.40	1.72
	**Hand–Wheel Interactions**	99.36	99.37	99.44	99.40	1.70
	**Driver Actions**	98.39	98.43	98.39	98.41	1.84
Face Camera Perspective	**Gaze Classification**	86.47	86.49	86.59	86.54	1.77
	**Driver Actions**	88.94	89.29	88.94	89.11	1.79
Hand Camera Perspective	**Hand–Wheel Interactions**	79.07	79.46	78.07	78.76	1.76
	**Driver Actions**	83.10	85.69	83.10	84.38	1.80
**Setup 2**						
Body Camera and Face Camera Perspectives	**Gaze Classification**	92.16	92.48	92.16	92.32	2.80
Body Camera and Hand Camera Perspectives	**Hand–Wheel Interactions**	92.37	92.21	92.37	92.28	2.72
Body Camera, Face Camera, and Hand Camera Perspectives	**Driver Actions**	92.97	93.43	92.97	93.20	2.97

**Table 9:** Summary of proposed model performance while testing on other datasets.

Dataset	Accuracy, %	Precision, %	Recall, %	F1-Score, %
**Driver Actions**				
**DMD**	92.97	93.43	92.97	93.20
**AUC_V2**	90.24	90.83	90.18	90.18
**SAM-DD**	89.19	89.46	89.70	89.70
**Gaze Classification**				
**DMD**	92.16	92.48	92.16	92.32
**AUC_V2**	91.05	91.21	91.15	91.18
**SAM-DD**	90.66	90.16	90.98	90.57
**Hand–Wheel Interactions**				
**DMD**	92.37	92.21	92.37	92.30
**AUC_V2**	90.06	90.14	90.26	90.21
**SAM-DD**	89.77	90.15	90.07	90.11

**Table 10:** Summary of proposed multi-task multi-perspective model testing results.

	Task	Accuracy, %	Precision, %	Recall, %	F1-Score, %	Inference Time, ms
**Setup 3**						
Body Cam, Face Cam & Hands Cam Perspectives	**Gaze Classification**	93.74	94.76	95.42	95.01	10.08
	**Hand–Wheel Interactions**					
	**Driver Actions**					

**Table 11:** Summary of multi-task and multi-perspective model results for predicting driver distraction compared to other studies on the DMD dataset.

Ref.	Input Type	Perspective	No. of Tasks.	Method	Accuracy, %
[34], 2024	Few-Shot Video	Face	17 (using a cellphone, distraction, yawning)	GPT-4V vision–language transformer, for:	
				Using a cellphone	90.9
				Distraction	91.0
				Yawning	98.2
				Model accuracy.	93.4
[30], 2022	Images	Body	5 (distraction)	AB-DLM: YOLOv5s + BiFPN	97.4 (precision)
[33], 2023	Time–surface event representation	Face	2 (distraction)	Sparse-Resnet	80.05
[32], 2024	Images	Body	10 (distraction)	Single-frameCLIP (vision transformer, CLIP)	82.65
[32], 2024	Video	Body	10 (distraction)	VideoCLIP (vision transformer, CLIP, S3D-Separatable 3D CNN)	98.44
[31], 2024	Images	Body and Face	20 (hands, gaze, actions, fatigue)	3DCNN (for hand–wheel interactions)	74.72
				I3D (for driver actions)	84.89
				SqueezeNet (for gaze)	89.47
				SqueezeNet (for fatigue)	96.32
				Model accuracy.	86.35
[52], 2024	Images	Body and Face	10 (distraction)	Weighted multi-task vision transformer	94.11
[29], 2024	Image	Body, Face, and Hands	10 (distraction)	ConvNet, multi-task ensemble learning	96.22
**This study**	**Video**	**Body, Face, and Hands**	**16** (hands, gaze, actions)	**3D Resnet-18 + BiLSTM**	**93.74**

**Table 12:** Summary of fused model results for predicting driver attentiveness state on the DMD dataset.

Threshold	Accuracy, %	Precision, %	Recall, %	F1_Score	ROC AUC
0.4	94.11	93.96	93.73	93.84	0.9563
**0.5**	**96.78**	**98.01**	**98.56**	**98.28**	**0.9563**
0.6	94.03	93.96	93.73	93.84	0.9563
0.7	93.12	87.19	87.02	87.09	0.9563
0.8	87.07	87.19	87.11	87.09	0.9563
0.9	83.33	83.86	83.86	83.35	0.9563

## Data Availability

Data is available upon request to the authors.
